# Correlation between matrix metalloproteinase expression and activation of the focal adhesion kinase signaling pathway in herpes stromal keratitis

**DOI:** 10.3892/etm.2013.1407

**Published:** 2013-11-13

**Authors:** TING CAO, YIQIAO XING, YANNING YANG, HAIFENG MEI

**Affiliations:** Department of Ophthalmology, Renmin Hospital of Wuhan University, Wuhan, Hubei 430060, P.R. China

**Keywords:** herpes stromal keratitis, matrix metalloproteinase, focal adhesion kinase pathway

## Abstract

The present study aimed to investigate the correlation between matrix metalloproteinase-2 (MMP-2) expression and activation of the focal adhesion kinase (FAK) signaling pathway in herpes stromal keratitis (HSK). The cornea of 24 BALB/c mice was infected with herpes simplex virus type 1 (HSV-1) to construct a model of HSK. Six additional mice served as negative controls. Immunohistochemical staining was used to detect FAK expression levels. Human corneal epithelial (HCE) cells cultured *in vitro* were infected with HSV-1 and the expression levels of MMP-2, FAK and phosphorylated-FAK (p-FAK) in HCE cells were detected using reverse transcription-polymerase chain reaction (RT-PCR), western blot analysis and immunohistochemistry at 2, 20 and 40 h following infection. In the HSK rat model, the corneal epithelial cells appeared deranged and the number of neutrophils and FAK-positive cells was significantly increased compared with that of the negative control group (P<0.05). Repeated measures analysis of variance of RT-PCR showed no significant differences in MMP-2 and FAK mRNA expression levels in the infected cells at various time points, and no significant differences between infected cells and the negative control group were observed. There was no interaction between groups and time points. Pairwise comparisons showed that MMP-2 and FAK mRNA expression levels were significantly increased in virus-infected cells compared with those of the control group. Over time, MMP-2 and FAK mRNA expression levels did not differ significantly in virus-infected cells or in control cells. Western blot analysis indicated no significant differences in p-FAK, FAK and MMP-2 expression levels between the infected and control cells at 2 h (P>0.05). Infected cells showed a significant increase in MMP-2 and p-FAK expression levels than that of the control cells at 20 and 40 h (P<0.05). p-FAK, FAK and MMP-2 expression levels in virus-infected cells at 2 h differed significantly from those at 20 and 40 h (P<0.05). Immunohistochemical staining results showed that a longer infection time was associated with an increased number of cells staining positive for MMP-2, FAK and p-FAK. Following HSV-1 infection of the corneal epithelium, the FAK signaling pathway was activated, resulting in increased secretion of MMP-2 in the corneal tissue and accelerated formation of corneal ulcers and necrotic lesions.

## Introduction

Herpes stromal keratitis (HSK), caused by herpes simplex virus type 1 (HSV-1) infection of the eye, is an eye disease that severely affects visual function, with a high blindness and relapse rate. Matrix metalloproteinases (MMPs) are a family of protein-cleaving enzymes that degrade the extracellular matrix (ECM) and basement membrane components. They are involved in a number of physiological processes, including damage repair, and in the pathological processes of specific diseases, for example tumor metastasis and keratopathy. A previous study (1) showed that, following HSV-1 infection of the cornea, MMP-2 (produced by corneal cells and corneal epithelial cells) plays an important role in the development of HSK-induced corneal ulceration. However, these observations only confirmed a change in MMP-2 expression levels and activity in the HSK model and the mechanisms by which HSK affects MMP-2 expression, secretion and activity, remain unclear.

Previous studies have shown that the secretion of MMPs may be regulated by activating multiple intracellular signaling pathways, including the focal adhesion kinase (FAK) signaling pathway (2). FAK is an important non-receptor protein tyrosine kinase that is important in intercellular adhesion and adhesion between cells and the ECM. Simultaneously, it is closely associated with embryonic development, cell cycle regulation and angiogenesis. Following activation, FAK undergoes a conformational change which results in the exposure of the catalytic domain, simultaneous to autophosphorylation and formation of phosphorylated-FAK (p-FAK) (3). After activation, FAK is widely involved in a variety of biological processes through multiple pathways, including the integrin pathway (4). Previous studies (5–8) have demonstrated that activation of FAK may result in the formation of the FAK-Src-p130Cas-Dock180 signaling complex and elevated activation of the c-Jun NH2-terminal kinase (JNK). Thereby, expression levels and activity of MMP is increased. We hypothesize that HSV-1 infection of corneal epithelial cells may activate FAK through the virus or via the release of cytokines by inflammatory cells, resulting in increased MMP levels in the corneal tissue and accelerated formation of corneal ulcers and necrotic lesions. The present study investigated the secretion of MMP-2, which is directly induced by HSV-1 infection, and its association with FAK and p-FAK expression, using immunohistochemical staining and molecular biology techniques to further explore the mechanisms of corneal ulceration in HSK.

## Materials and methods

### Construction of an experimental animal model of HSK

The Institutional Animal Care and Use Committee of Wuhan University (Wuhan, China) approved the study protocol. Animal care guidelines comparable to those published by the Institute for Laboratory Animal Research (Guide for the Care and Use of Laboratory Animals, 8th edition) were followed. Thirty female BALB/c mice (weight, 60–80 g; age, 6–8 weeks), with normal bilateral anterior segment examination results, were provided by the Zhongnan Hospital Experimental Animal Center, Wuhan University. The animals were randomly divided into five groups (n=6 per group), the negative control group and four experimental groups. Mice in the experimental groups were anesthetized by intraperitoneal injection of sodium pentobarbital (50 mg/kg). Under a slit lamp microscope (SL 115 Slit Lamp; Carl Zeiss, Oberkochen, Germany), cross-shaped corneal epithelial scratches were made with a 5-ml syringe needle in the right eyes of the animals in the experimental groups to the depth of the Bowman's layer within the limbus. One drop of 5 μl HSV-1 suspension (Institute of Virology, College of Medicine, Wuhan University; 10^5^ PFU) was placed onto the cornea and allowed to remain for 20 sec. Commercially-available chloramphenicol eye drops were applied to the eyes daily, and one experimental group was sacrificed each day at 1, 7, 14 and 28 days following infection. The eyes were collected and fixed in 4% formalin solution for 6 h. The controls were sacrificed 28 days following infection by cervical vertebra dislocation and the eyes were collected and fixed as described.

### Immunohistochemical staining

Following fixation, each eye was embedded in paraffin with the cornea facing to the side to facilitate sectioning. The cornea was sectioned into 5 μm-thick slices. The slices were dewaxed and washed with phosphate-buffered saline (PBS; pH 7.4) three times for 5 min each. Slices were placed in boiling water containing EDTA (pH 8.0) under a high pressure for 10 min to retrieve the antigen. The sections were then placed in cold water and cooled to room temperature. Slices were incubated in 3% hydrogen peroxide solution at room temperature for 10 min to block endogenous peroxidase and then washed with PBS (pH 7.4) and incubated overnight at 4°C with anti-FAK antibody (1:100; Wuhan Boster Biological Technology, Ltd., Wuhan, China). The two-step anti-rabbit/mouse universal immunohistochemistry kit (EnVision Detection Systems Peroxidase/DAB, Rabbit/Mouse; DakoCytomation, Glostrup, Denmark) was used for color reaction. The slices were observed and images were captured under a microscope (UB102i; Chongqing UOP Photoelectric Technology, Chongqing, China).

### Cell count

FAK-positive cells were counted at the periphery and center of the cornea using 10×10 grid under a high-powered field (magnification, ×450) at 1, 7, 14 and 28 days following infection in the experimental group and the results were compared with the corneal slices of the negative control group. We selected 6 slices from each group (total 30 slices) to conduct the cell count.

### Primary culture of human corneal epithelial (HCE) cells

The Institutional Review Board of Wuhan University approved the use of HCE cells. The tissue block culturing method was used. Tissue blocks were obtained from the remaining donor corneas following penetrating keratoplasty. Under a surgical microscope (OPMI Lumera 700; Carl Zeiss), the endothelial cell layer and matrix layer were peeled off and inoculated in a Petri dish with the epithelial cell layer at the top. Dulbecco's modified Eagle's medium (DMEM; HyClone, Waltham, MA, USA) solution was used. The samples were cultured in an incubator with 5% CO_2_ at 37°C for 72 h. The second-generation cells were collected for immunohistochemistry with mouse anti-human cytokeratin 3 monoclonal antibody to confirm the identity of the cell line (Santa Cruz Biotechnology, Inc., Santa Cruz, CA, USA). Cells of passages 2–4 were collected for further experiments.

### Infection of HCE cells with HSV-1

When HCE cells reached 70–90% confluence the medium was discarded. The HSV-1 (F strain) suspension was inoculated using an optimal multiplicity of infection (MOI) of 5. Samples were placed in an incubator for 1 h and the flask was shaken once every 15 min to ensure the cells evenly absorbed the virus suspension. UltraCULTURE Serum-free Medium (12–725F; LONZA Inc., Basel, Switzerland) was added to the negative control group and the cells used were non-infected cells. The suspension was discarded after 1 h, serum-free medium was added and the samples were cultured in an incubator with 5% CO_2_ at 37°C. Cells positive for enhanced green fluorescent protein (EGFP) represented cells successfully infected with HSV-1. The number of infected cells and the total number of cells in the same field were counted. The infection efficiency (%) was calculated using the following formula: Number of EGFP-positive cells/total number of cells × 100.

### Detection of MMP-2 and FAK mRNA expression using reverse transcription-polymerase chain reaction (RT-PCR)

HCE cells were collected at 2, 20 and 40 h following HSV-1 infection and total RNA was extracted. The cDNA was obtained through RT of random primers and was used for PCR amplification. According to the mRNA sequences of β-actin, FAK and MMP-2 in GenBank, specific amplification primers were designed using Primer 5 software (Primer Premier 5.0; Premier Biosoft Inc., Canada) as follows: β-actin (accession no. NM_001101) upstream, 5′-GTCCACCGCAAATGCTTCTA-3′ and downstream 5′-TGCTGTCACCTTCACCGTTC-3′ (length, 190 bp); FAK (accession no. NM_005607.3) upstream, 5′-CCCTATGGTGAAGGAAGTCG-3′ and downstream, 5′-TGCCATCTCAATCTCTCGGT-3′ (length 106 bp); and MMP-2 (accession no. NM_004530.4) upstream, 5′-AGTGACGGAAAGATGTGGTGTG-3′ and downstream, 5′-CTTGGTGTAGGTGTAAATGGGTG-3′ (length 182 bp). Following electrophoresis of the RT-PCR products, gels were scanned using the computer image analyzer (AlphaEaseFC software; Alpha Innotech Corporation, San Leandro, CA, USA) and the gray values of the bands were analyzed using the electrophoresis gel image analysis system (AlphaEaseFC, Genetic Technologies, Inc., Miami, FL, USA). The mean gray values of the target fragment and the internal reference were compared in order to calculate the ratio.

### Detection of MMP-2, FAK and p-FAK protein expression using western blot analysis

Cell lysis buffer (100 μl) was added to a Petri dish at 2, 20 and 40 h after HSV-1 infection and the cells were collected following 30 min of lysis on ice. Cells were sonicated and centrifuged at 12,000 rpm for 5 min at 4°C (Labofuge 400R; Thermo Fisher Scientific, Rockford, IL, USA). The supernatant was collected, the protein content of each sample was measured with the spectrophotometer using the BAC method and the concentration of the sample for loading was determined. SDS-PAGE was performed for protein detection and the proteins were transferred to nitrocellulose membranes (LC2000; Invitrogen, Carlsbad, CA, USA). Nonspecific immunoglobulin binding was blocked with skimmed milk and bovine serum albumin for 1 h. The filter membrane was incubated with rabbit anti-human MMP-2, FAK and p-FAK polyclonal antibodies (Wuhan Boster Biological Technology, Ltd.) at 4°C overnight. Following rinsing with PBS buffer, the membrane was incubated with the corresponding horseradish peroxidase-conjugated secondary antibody (Wuhan Boster Biological Technology, Ltd.) at room temperature for 1 h. The enhanced chemiluminescence method was used to detect protein expression [BeyoECL Plus kit (Beyotime, P0018); Beyotime Institute of Biotechnology, Shanghai, China] and images were captured of the membranes. The gel image processing system was used to analyze the molecular weight and net optical density values of the target bands.

### Detection of MMP-2, FAK and p-FAK protein expression using immunohistochemical staining

Cells were seeded in 24-well plates at 2, 20 and 40 h following infection and fixed with 4% paraformaldehyde for 30 min. The samples were treated with Triton X-100 for 15 min and blocked with 3% H_2_O_2_-methanol for 15 min. Rabbit anti-human MMP-2, FAK and p-FAK polyclonal antibodies were then added. The two-step anti-rabbit/mouse universal immunohistochemistry kit was used for staining development. The samples were observed under a microscope and images were captured.

### Statistical analysis

The experimental data are expressed as the mean ± standard deviation. Repeated measures analysis of variance was performed. P<0.05 was considered to indicate a statistically significant difference. Data was analyzed using SPSS software, version 16.0 (SPSS, Inc., Chicago, IL, USA).

## Results

### Morphological changes following HSV-1 infection

**Figure 1:**
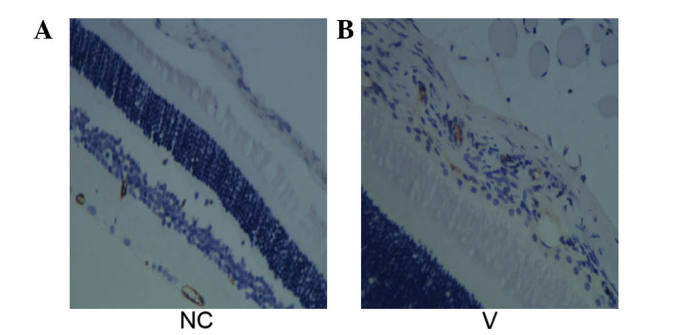
Hematoxylin and eosin staining showed morphological changes of the cornea in the experimental and control groups (magnification, ×400). (A) In the NC group, tight and orderly corneal epithelium with no neutrophils in the epithelium or stroma was observed. (B) In the V group, disordered cell arrangement, numerous neutrophils and matrix edema with eventual necrosis in the epithelial cells was observed. NC, normal control group; V, infected group.

The corneal epithelium of the negative control group was orderly and tightly arranged. No neutrophils were observed in the epithelium or stroma. Epithelial edema and neutrophil infiltration were observed on the first day following HSV-1 infection. Over time, vacuolar degeneration and necrosis gradually occurred in epithelial cells. Cell arrangement was disordered, matrix edema was present and the number of neutrophils increased (Fig. 1).

### Tissue localization of FAK and cell count

**Figure 2:**
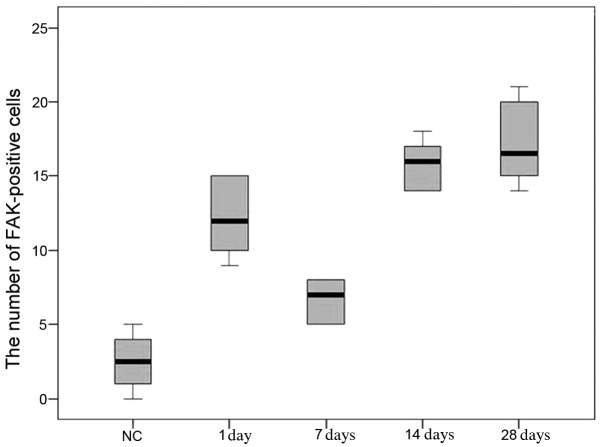
Number of FAK-positive cells in the control group and at 1, 7 14 and 28 days following infection. In the corneal epithelium, the number of FAK-positive cells increased significantly 1 day after HSV-1 infection and declined on day 7. The number increased again on days 14 and 28, differing significantly from the remaining groups (P<0.05). FAK, focal adhesion kinase; HSV-1, type 1 herpes simplex virus.

In the cornea of the negative control group, FAK-positive cells were located mainly near the basement membrane in the corneal epithelial cells. In the corneal epithelium, the number of FAK-positive cells increased significantly 1 day following HSV-1 infection compared with that of the negative control group (P<0.05); and the number of FAK-positive cells declined on day 7 compared with that on day 1 (P<0.05). The number increased again on days 14 and 28, differing significantly from that of the negative control group and the four groups with HSV-1 infection (P<0.05). However, the number of FAK-positive cells on days 14 and 28 were not significantly different (P>0.05) (Fig. 2).

### Cell number

**Figure 3:**
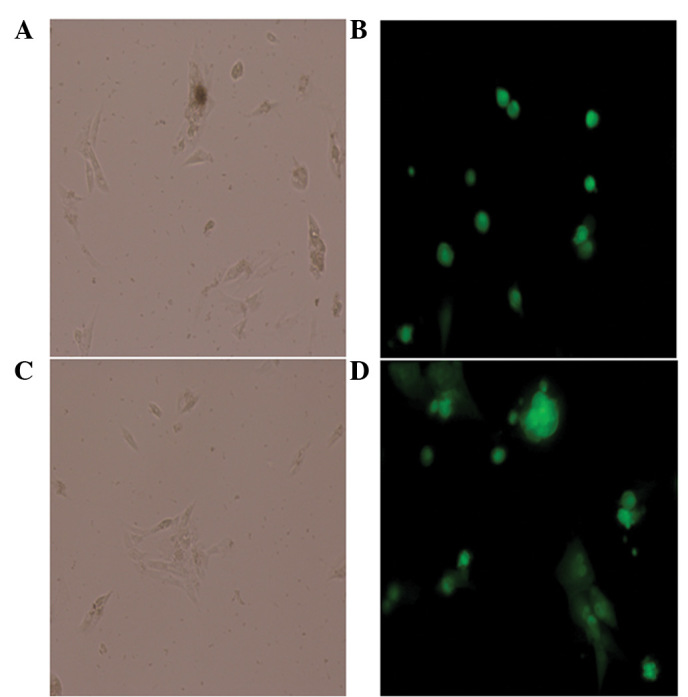
Efficiency of HSV-1 infection in HCE cells. HCE cells infected with HSV-1 virus in (A and C) the bright field and (B and D) the fluorescence field, when MOI=5. The proportion of GFP-positive cells among all the cells in five randomly selected fields were calculated and the infection efficiency was 92%. HSV-1, type 1 herpes simplex virus; HCE, human corneal epithelial; MOI, multiplicity of infection.

The number of cells marked with green fluorescence and the total number of cells in the same field were counted under a phase contrast fluorescence inverted microscope (Fig. 3). The virus infection efficiency was 92% when the MOI was 5, which met the experimental requirement.

### MMP-2 and FAK mRNA expression levels in HCE cells detected by RT-PCR

**Figure 4:**
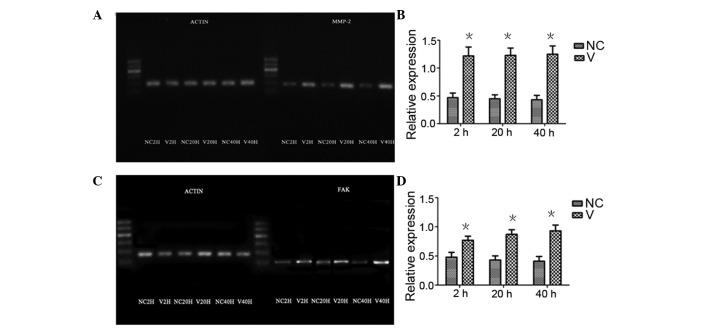
Reverse transcription-polymerase chain reaction detection of changes in MMP-2 and FAK mRNA expression levels in HCE cells following HSV-1 infection. (A) MMP-2 mRNA expression levels in HCE cells following HSV-1 infection. (B) Histogram of the relative expression of MMP-2 mRNA in V and NC cells in (A). Compared with that of the NC group, MMP-2 mRNA expression levels were significantly increased in the V group. (C) FAK mRNA expression levels in HCE cells following HSV-1 infection. (D) Histogram of the relative expression of FAK mRNA in V and NC cells in (C). FAK mRNA expression levels were significantly increased in the V group ^*^P<0.05, vs. NC group. MMP-2, matrix metalloproteinase-2; FAK, focal adhesion kinase; HCE, human corneal epithelial; HSV-1, type 1 herpes simplex virus; V, infected with HSV-1; CN normal control.

The MMP-2 and FAK mRNA expression levels in the HCE cells detected by RT-PCR at 2, 20 and 40 h following HSV-1 infection, are shown in Fig. 4 and Tables I and II. The repeated measures analysis of variance revealed no significant differences in MMP-2 and FAK mRNA expression levels at different time points (F=0.968, P=0.436). Both MMP-2 and FAK mRNA expression levels differed significantly between infected cells and non-infected cells (F=47.649, P=0.000), with no interaction between groups and time points (F=0.757, P=0.536). Pairwise comparisons showed a greater mRNA expression in the virus-infected cells than in non-infected cells at each time point (P<0.01). Over time, virus-infected and non-infected cells showed no significant differences in mRNA expression levels of MMP-2 and FAK.

### p-FAK expression in HCE cells detected by western blot analysis

**Figure 5:**
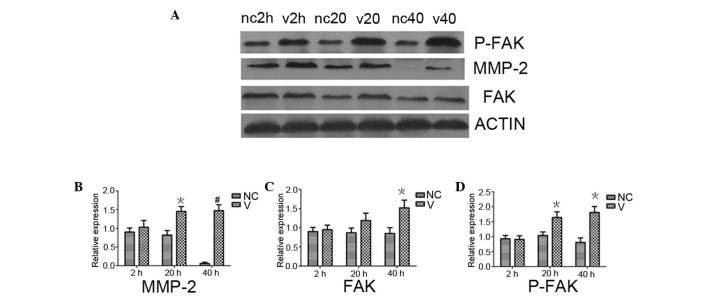
Western blot analysis of MMP-2, p-FAK and FAK protein expression levels in HCE cells following HSV-1 infection. (A) MMP-2, p-FAK and FAK protein expression levels in HCE cells following HSV-1 infection. Histogram of the relative expression of (B) MMP-2, (C) FAK, and (D) p-FAK in V and NC cells in (A). V cells showed an increasing trend in protein expression of MMP-2, p-FAK and FAK with increased infection time. ^*^P<0.05 and ^#^P<0.01, vs. NC group. MMP-2, matrix metalloproteinase-2; phosphorylated-FAK, focal adhesion kinase; HCE, human corneal epithelial; HSV-1, type 1 herpes simplex virus; V, infected with HSV-1; CN normal control.

p-FAK expression in HCE cells detected by western blot analysis at 2, 20 and 40 h following HSV-1 infection, are shown in Fig. 5D. No statistically significant differences were observed between infected and non-infected cells 2 h after infection (P>0.05). At 20 and 40 h following infection, p-FAK expression differed significantly between infected and non-infected cells (P<0.05). p-FAK expression levels in the infected cells 2 h after infection was significantly different than that at 20 and 40 h (P<0.05). Over time, p-FAK expression of non-infected cells did not change significantly (P>0.05). FAK expression levels in HCE cells at 2, 20 and 40 h following HSV-1 infection (Fig. 5C) was in line with the aforementioned p-FAK expression. MMP-2 expression levels (Fig. 5B) in HCE cells at 2, 20 and 40 h following HSV-1 infection showed no statistically significant difference between infected cells and non-infected cells 2 h after infection (P>0.05). At 20 and 40 h, MMP-2 expression levels were significantly greater in infected cells (P<0.05 and P<0.01, respectively) than the non-infected cells. At 2 h, MMP-2 expression levels in infected cells was significantly lower than that at 20 and 40 h (P<0.05). At 40 h, MMP-2 expression levels in non-infected cells was significantly different from that in the same cells at 2 and 20 h after infection (P<0.05).

### Cells with positive immunohistochemical staining for MMP-2, FAK and p-FAK

**Figure 6:**
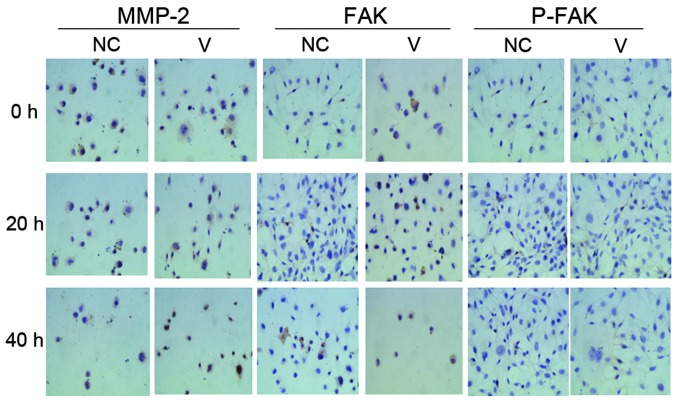
Detection of MMP-2, FAK and p-FAK protein expression using immunohistochemical staining (magnification, ×400). Cells with positive immunohistochemical staining showed blue nuclei. The cytoplasm was stained a specific brownish yellow. As infection time increased, the number of cells which stained positive increased. MMP-2, matrix metalloproteinase-2; FAK, focal adhesion kinase; p-FAK, phosphorylated-FAK.

Cells with positive immunohistochemical staining for MMP-2, FAK and p-FAK showed blue nuclei. The cytoplasm was stained a specific brownish yellow (Fig. 6). As infection time increased, the number of positively stained cells increased.

## Discussion

The present study demonstrated the model construction of HSK in rats. We observed morphological changes in cells of the epithelial layer during viral infection and increased neutrophil infiltration in the corneal stroma, confirming that HSV-1 infection induced leukocyte migration into the limbal blood vessels and activated the inflammatory response.

In the negative control group, several FAK-positive cells were identified in the corneal epithelium of mice suggesting that FAK is important in maintaining the normal physiological function of cells. On day 1 following HSV-1 infection, FAK-positive cells increased in the corneal epithelium, but the number of FAK-positive cells decreased 7 days after infection. The number of FAK-positive cells increased again on day 14 and 28 after infection in an upward step-wise fashion. The pattern of FAK expression levels was similar to that of MMP-2 in the cornea of an experimental animal model of HSK by Yang *et al*(1). These results indicate that MMP-2 is important in the development of corneal stromal ulcers.

A previous study (9) also showed that p-FAK is important in the transfer of viral capsids to the host nuclear pore complex. Therefore, we hypothesized that in the initial stage of HSV-1 infection, the main purpose of activating a large amount of FAK protein is to respond to viral replication and invasion of the host cells. When the host immune function is activated, viral replication and invasion are decreased, therefore, FAK activation is also reduced. In this case, the viruses that have invaded the host cell destroyed these cells, and the large amount of FAK protein may activate the expression of cytokines, for example MMP-2, which further assists in the formation of corneal stromal ulcers.

The present study successfully infected HCE cells with HSV-1 *in vitro* and detected MMP-2 mRNA and protein expression levels at the early stages of infection. The results showed that mRNA expression levels 2 h following infection were significantly greater than that of normal cells, and continued to increase up to 40 h after infection. Increases in MMP-2 protein expression were slower and were not significantly different from that of the normal cells 2 h after infection, but increased with time from 20 to 40 h. These results are consistent with the findings of previous studies. Using *in vivo* animal models of HSK, Yang *et al*(10) indicated that MMP-2 protein expression peaked between days 2 and 14 following infection, and the expression was mainly located at the base of the epithelial cells and in the superficial stroma. After 2 days, the epithelium healed and protein expression levels declined until inflammatory cells synthesized a large number of MMP-2 proteins again following formation of stromal ulcers. The present study validated the change in MMP-2 protein expression in corneal epithelial cells within 2 days of infection, indicating that MMP-2 synthesized by epithelial cells at the early stages of infection is important in the formation of corneal stromal ulcers.

Cheshenko *et al*(9) found that at the early stages of HSV-1 infection of cervical epithelial cells, p-FAK is important in the transfer of viral capsids to the host cell nuclear pore complex. Therefore, silencing the FAK gene can reduce the viral infection rate by 90%. In the present study, the authors observed that FAK mRNA expression 2 h after infection was significantly higher than that in the normal cells and expression continued to increase up to 40 h. The levels of protein expression of FAK and p-FAK did not differ significantly from those of normal cells 2 h after infection. The expression of these proteins increased significantly with time from 20 to 40 h and the change in expression was similar to that of MMP-2 protein. Hsia *et al*(8) identified that FAK activation may result in the formation of the FAK-src-p130Cas-Dock180 signaling complex and an increased level of Rac and JNK activation, thereby stimulating the expression and enhancing the activity of MMP. Heiligenhaus *et al*(11) previously performed gelatin zymography and identified that following inoculation the corneas of mice with HSV-1, and the activity levels of MMP-2, −8 and −9 in the corneal tissue were significantly elevated on day 2. The results of present study indicated that at the early stages of HSV-1 infection of cultured HCE cells *in vitro*, p-FAK not only participates in the transfer of viral capsids into the nucleus but also activates MMP-2 expression, resulting in the formation of corneal stromal ulcers. Following HSV-1 infection of the corneal epithelium, the FAK signaling pathway was activated, resulting in increased secretion of MMP-2 in the corneal tissue and accelerated formation of corneal ulcers and necrotic lesions. The present study investigated the secretion of MMP-2, which is directly induced by HSV-1 infection, and its association with FAK and P-FAK expression. P-FAK plays an important role in MMP-2 activation when the HSV-1 infects cells. Further study is required on the mechanisms of corneal ulceration in HSK.

## Figures and Tables

**Table I:** Relative gray values of matrix metalloproteinase-2 mRNA electrophoretic bands in infected and non-infected cells at various time points, obtained from RT-PCR.

Parameters	2 h	20 h	40 h	F statistic	P-value
Non-infected cells	0.47±0.05	0.45±0.02	0.43±0.07	0.771	0.485
Infected cells	1.22±0.02	1.23±0.09	1.25±0.10	0.190	0.830
t-test	31.142	18.918	15.021	-	-
P-value	0.000	0.000	0.000	-	-

**Table II:** Relative gray values of the focal adhesion kinase mRNA electrophoretic bands in infected and non-infected cells at various time points, obtained from RT-PCR.

Parameters	2 h	20 h	40 h	F statistic	P-value
Non-infected cells	0.40±0.03	0.43±0.06	0.41±0.08	0.321	0.731
Infected cells	0.87±0.05	0.87±0.04	0.93±0.04	3.610	0.079
t-test	18.024	13.644	13.000	-	-
P-value	0.000	0.000	0.000	-	-

## References

[1] Yang YN, Bauer D, Wasmuth S, Steuhl KP, Heiligenhaus A (2003). Matrix metalloproteinases (MMP-2 and 9) and tissue inhibitors of matrix metalloproteinases (TIMP-1 and 2) during the course of experimental necrotizing herpetic keratitis. Exp Eye Res.

[2] Budagian V, Bulanova E, Orinska Z, Pohl T, Borden EC, Silverman R, Bulfone-Paus S (2004). Reverse signaling through membrane-bound interleukin-15. J Biol Chem.

[3] Mitra SK, Hanson DA, Schlaepfer DD (2005). Focal adhesion kinase: in command and control of cell motility. Nat Rev Mol Cell Biol.

[4] Cohen LA, Guan JL (2005). Mechanisms of focal adhesion kinase regulation. Curr Cancer Drug Targets.

[5] Hu B, Jarzynka MJ, Guo P, Imanishi Y, Schlaepfer DD, Cheng SY (2006). Angiopoietin 2 induces glioma cell invasion by stimulating matrix metalloprotease 2 expression through the alphavbeta1 integrin and focal adhesion kinase signaling pathway. Cancer Res.

[6] Segarra M, Vilardell C, Matsumoto K, Esparza J, Lozano E, Serra-Pages C, Urbano-Márquez A, Yamada KM, Cid MC (2005). Dual function of focal adhesion kinase in regulating integrin-induced MMP-2 and MMP-9 release by human T lymphoid cells. FASEB J.

[7] Akahane T, Akahane M, Shah A, Connor CM, Thorgeirsson UP (2004). TIMP-1 inhibits microvascular endothelial cell migration by MMP-dependent and MMP-independent mechanisms. Exp Cell Res.

[8] Hsia DA, Mitra SK, Hauck CR (2003). Differential regulation of cell motility and invasion by FAK. J Cell Biol.

[9] Cheshenko N, Liu W, Satlin LM, Herold BC (2005). Focal adhesion kinase plays a pivotal role in herpes simplex virus entry. J Biol Chem.

[10] Yang Y, Xing YQ, Dirk B, Arnd H (2002). Localization of matrix metalloproteinase-2 in experimental herpes simple virus keratitis. Med J Wuhan Univ.

[11] Heiligenhaus A, Li HF, Yang Y, Wasmuth S, Steuhl KP, Bauer D (2005). Transplantation of amniotic membrane in murine herpes stromal keratitis modulates matrix metalloproteinases in the cornea. Invest Ophthalmol Vis Sci.

